# Intracerebroventricular infusion of donepezil prevents cardiac remodeling and improves the prognosis of chronic heart failure rats

**DOI:** 10.1186/s12576-020-00739-0

**Published:** 2020-02-17

**Authors:** Meihua Li, Can Zheng, Toru Kawada, Masashi Inagaki, Kazunori Uemura, Masaru Sugimachi

**Affiliations:** Department of Cardiovascular Dynamics, National Cerebral and Cardiovascular Center, Osaka, Japan

**Keywords:** Donepezil, Intracerebroventricular infusion, Cardiac remodeling, Chronic heart failure, Survival rate

## Abstract

Oral administration of donepezil, a centrally acting acetylcholinesterase inhibitor, improves the survival of rats with chronic heart failure (CHF). The mechanisms of cardioprotective effects of donepezil, however, remain totally unknown. To elucidate potential mechanisms, we examined whether central microinfusion of donepezil would exert cardioprotection. Intracerebroventricular microinfusion pumps with cerebroventricular cannula were implanted in rats with myocardial infarction. The rats were randomly divided into central saline treatment (CST) and central donepezil treatment (CDT) groups. We evaluated cardiac remodeling and function after a 6-week treatment and examined the 160-day survival rate. Compared to the CST, the CDT markedly improved the 160-day survival rate (68% vs. 32%, *P* = 0.002) through the prevention of cardiac remodeling and the lowering of plasma catecholamine, brain natriuretic peptide, and angiotensin II. These results suggest that the central mechanism plays an important role in the cardioprotective effects of donepezil.

## Background

Despite significant advances in therapies and prevention [1], chronic heart failure (CHF) is a major and growing public health problem worldwide [2]. Most effective CHF drugs, including β-blockers [3–5], angiotensin-converting enzyme inhibitors [6, 7], and angiotensin II receptor blockers [8], counteract sympathetic neurohumoral activation. Nevertheless, mortality and morbidity are still high, and quality of life remains low among patients with CHF. The decreased parasympathetic function is also an independent risk factor after acute myocardial infarction (MI) [9, 10], but few studies have investigated treatment alternatives to combat parasympathetic dysfunction [11]. As an alternative therapeutic strategy, we have demonstrated that parasympathetic activation via electrical vagal nerve stimulation markedly improved the long-term survival of CHF rats [12]. We have also demonstrated that possible modulation of parasympathetic function by oral administration of donepezil prevented the progression of cardiac remodeling and improved the long-term prognosis in CHF rats with extensive MI [13, 14]. However, the mechanisms underlying these cardioprotective effects afforded by oral administration of donepezil remain unknown.

There have been no reports to prove the improved survival of CHF animals other than treated with donepezil. Other peripheral cholinesterase inhibitors seem less effective in improving survival [15]. Pyridostigmine, a peripheral acetylcholinesterase inhibitor, exerted a beneficial effect on CHF rats, but it did not improve survival [16, 17]. Donepezil is a centrally long-acting reversible acetylcholinesterase inhibitor [18, 19] and is prescribed orally to patients with Alzheimer's disease or vascular dementia to increase the level of central acetylcholine (ACh) [20]. Focusing on the capability of donepezil to reach the central nervous system [21–23], we hypothesized that oral administration of donepezil may improve CHF through central effects. The present study aimed to examine whether localized central microinfusion of donepezil, with virtually no systemic distribution, prevents cardiac remodeling and dysfunction and improves long-term survival in CHF rats. If the central microinfusion of donepezil fails to show cardioprotective effects, our hypothesis could be rejected.

## Materials and methods

### Animals

The care of animals and all animal experiments were performed in strict accordance with the Guide for the Care and Use of Laboratory Animals published by the US National Institutes of Health (NIH Publication No. 85-23, revised 1996), and the Guiding Principles for the Care and Use of Animals in the Field of Physiological Sciences, which have been approved by the Physiological Society of Japan. All protocols were reviewed and approved by the Animal Subject Committee in the National Cerebral and Cardiovascular Center.

### Experimental design

**Fig. 1:**
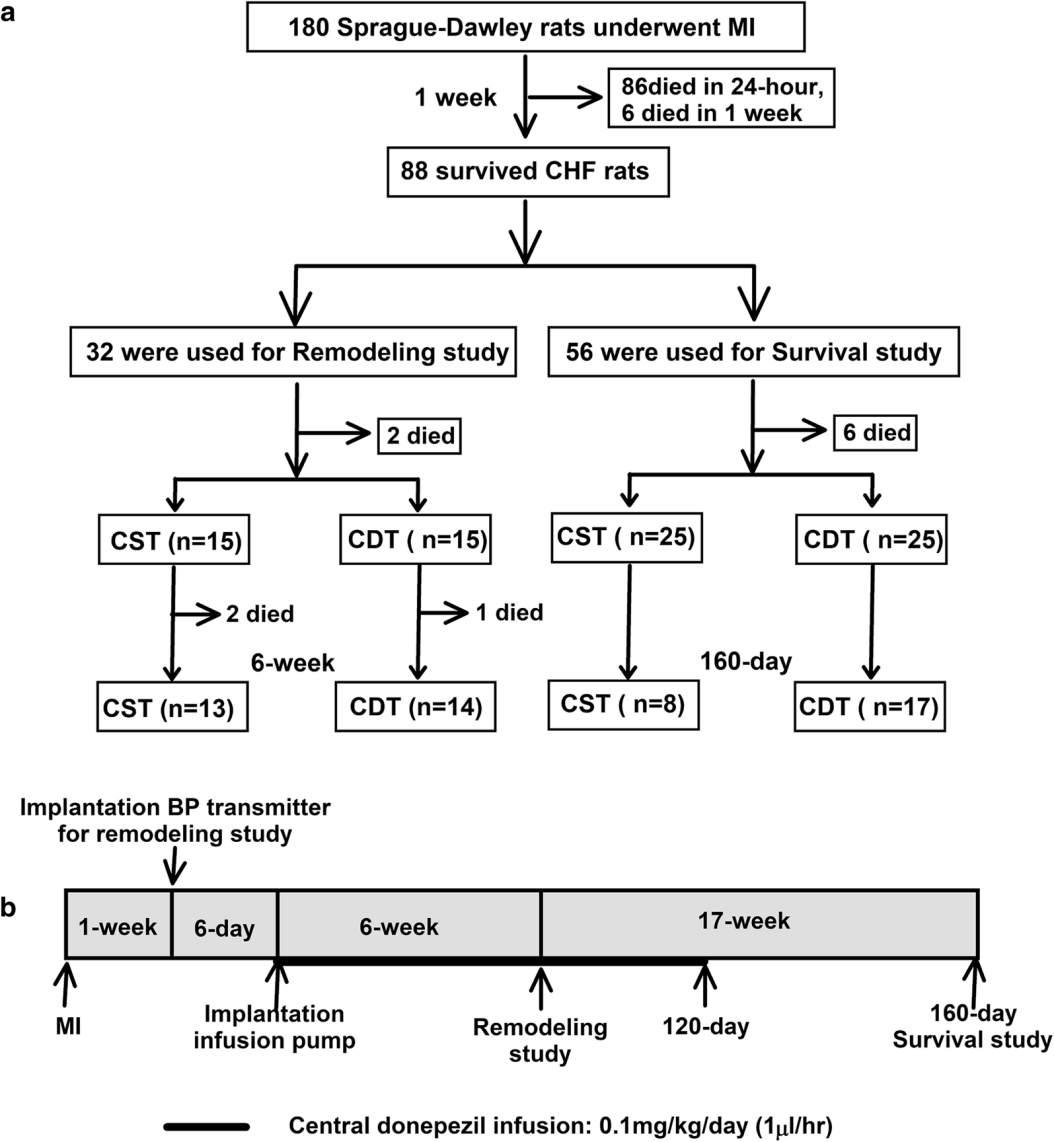
Experimental design and timeline. **a** Experimental design of central donepezil infusion therapy in chronic heart failure (CHF) rats with myocardial infarction (MI). **b** Experimental events and timeline of the remodeling study and survival study. *BP* blood pressure, *CDT* central donepezil treatment, *CST* central saline treatment

As shown in Fig. 1a, 88 permanent MI-induced CHF rats were used in this study. We performed different protocols for the remodeling study (*n* = 32) and survival study (*n* = 56) described below.

### CHF model

Permanent MI (infarct size ≥ 40%) was induced by occluding the proximal left coronary artery in 180 male 8-week-old Sprague-Dawley rats (body weight: 250–280 g; SLC, Hamamatsu, Japan) as described previously [12–14]. Slightly less than 50% of the animals with extensive MI survived after 1 week. We confirmed the infarct size by postmortem examinations.

### Telemetric long-term hemodynamic measurements

One week after inducing permanent extensive MI, we implanted blood pressure (BP) transmitters (TA11PA-C40, DSI, St. Paul, MN, USA) into the 32 MI-induced CHF rats to monitor their daily mean BP (MBP) and heart rate (HR) in the remodeling study. The Teflon tube portion of the BP transmitter was cannulated into the abdominal aorta. The pressure signal was acquired at a 500-Hz sampling rate, and the calculated MBP and HR data were recorded intermittently (for 5 s every 5 min) in the freely moving animals.

### Intracerebroventricular microinfusion

On the 13th day after induction of MI, the surviving rats were placed in a stereotaxic head holder. One hole was made for a steel cannula aimed at the right lateral cerebral ventricle at the coordinates: anteroposterior 0.8 mm behind the bregma, lateral 1.4 mm from the midline, and depth 4.0–4.5 mm from the skull surface. Two screws were anchored for dental cement fixation. An Alzet brain infusion kit 2 (cannula) was used with an iPrecio^®^ microinfusion pump (Primetech, Inc. Tokyo, Japan). In a preliminary test, we checked whether the drug was accurately being injected into the lateral ventricle using a blue dye. Donepezil or vehicle (saline) was administered at a rate of 1.0 μL/h. Donepezil (SIGMA-Aldrich, USA) was dissolved in saline to a dosage of 0.1 mg/kg/day, which is 1/50 of the oral dose used in a previous study [13]. An area under the curve of the blood concentration of donepezil is approximately three times higher for an intravenous administration than for an oral administration in rats [22]. Hence, the intravenous administration at 1.67 mg/kg/day would be comparable to the oral administration at 5 mg/kg/day. To reduce a possible systemic effect during the central donepezil infusion, the dosage was set to less than 1/10 of the putative dosage of the intravenous administration. Although the selection of the dosage was empirical, we confirmed that this dosage did not induce apparent acute systemic effects on hemodynamics in a preliminary study.

### Remodeling study

Thirty CHF rats equipped with a BP transmitter and central injection pump were randomly assigned to central saline treatment (CST, *n* = 15) or central donepezil treatment (CDT, *n* = 15) groups. Donepezil treatment was continued for 6 weeks (Fig. 1b).

#### Hemodynamic measurements under anesthesia

At the end of the 6-week treatment, we conducted an acute hemodynamic study in the surviving CHF rats (CST, *n* = 13; CDT, *n* = 14) under anesthesia (3% for induction, 1.2% for surgery, and 0.6% halothane during data recordings). Left ventricular (LV) pressure (LVP) was measured using a 2-Fr catheter-tip micromanometer (SPC-320, Millar Inc. Houston, TX, USA) through the right common carotid artery. LV end-diastolic pressure (LVEDP) was determined from LVP. The maximum positive d*p*/d*t* of LVP (LV + d*p*/d*t*_max_) and maximum negative d*p*/d*t* of LVP (LV − d*p*/d*t*_max_) was calculated. Right atrial pressure (RAP) was measured using a fluid-filled catheter (PE50) and transducer (DX-200, NIHON KOHDEN, Tokyo, Japan) through the right jugular vein. Cardiac index (CI) was defined as cardiac output/body weight. Cardiac output was an integral of ascending aortic flow measured using a transonic flow probe (T206 flow probes #2.5 SB1014, Transonic Systems Inc. Ithaca, NY, USA). All signals were digitized at a rate of 500 Hz for 1–2 min. After the hemodynamic measurements were completed, blood samples were collected from the carotid artery and were divided into ethylenediaminetetraacetic acid tubes containing aprotinin for inhibiting proteinase activity. Plasma samples were obtained after centrifugation (3000 rpm, 20 min) at 4 °C and were then separately stored at − 80 °C until the assays were conducted. Finally, the experimental animals were killed by an overdose of intravenous sodium pentobarbital (100 mg/kg). The heart was rapidly excised, the blood was rinsed off, and they were then weighed and sliced with a stainless-steel slicer for subsequent determination of infarct size.

#### Neurohumoral measurements

Plasma catecholamine concentrations were measured using high-performance liquid chromatography with electrochemical detection after alumina adsorption. Plasma concentrations of brain natriuretic peptide (BNP), arginine vasopressin (AVP), and angiotensin II (ANG II) were determined using enzyme-linked immunosorbent assay kits (BNP-32 Enzyme Immunoassay Kit, Peninsula Lab; arg8-Vasopressin Enzyme Immunoassay Kit, Assay Designs; Angiotensin II Enzyme Immunoassay Kit, SPI Bio).

#### Immunohistochemistry

Transmural blocks of biventricular myocardium obtained from the sliced heart were immersed in a fixative containing 4% paraformaldehyde and a 0.1 mol/L phosphate buffer (pH 7.4), embedded in paraffin, and were sectioned at a thickness of 4 µm. The sections were deparaffinized, placed in citrate buffer, and heated in an autoclave for 20 min at 121 °C to enhance specific immune staining. The sections were then incubated overnight with rabbit anti-von Willebrand factor (vWF) polyclonal antibody (1:200 dilution; Dako) at 4 °C and were then incubated for 2 h in Alexa 633-conjugated goat anti-rabbit IgG (1:100 dilution; Molecular Probes) at room temperature for the micro-vessel analysis [14, 24]. The fluorescence of Alexa 633 was observed under a fluorescent scanning microscope system (BZ-9000, Keyence, Japan). Capillary vessels in the peri-infarct area (1.0-mm bands next to the scar), excluding the scar region, were counted under a fluorescent scanning microscope system at 20× magnification. Data obtained from the high-power fields (8 areas for each rat) were averaged and expressed as the number of capillary vessels.

#### Determination of infarct size and fibrosis

Biventricular sections (4-µm thick) from the basal, middle and apical portions were stained using Masson’s trichrome method. Histological images were digitized using a frame grabber and then were analyzed. The infarct size was calculated from the three slices by dividing the sum of the endocardial lengths of the infarcted regions by the sum of the endocardial circumferences. Next, we randomly selected Masson’s trichrome stained slide samples and evaluated for areas of cardiac fibrosis. The extent of cardiac fibrosis was evaluated using a light microscope at 20× magnification. The area of fibrosis was calculated from 4 high-power fields in the non-infarcted septum and peri-infarcted area in each heart. The area of perivascular fibrosis was calculated from the coronary arteries with an inner diameter of 50–100 µm in the non-infarcted area in each heart.

### Survival study

To examine the outcome of central donepezil therapy, we analyzed the 160-day mortality rates for the two groups of CHF rats (CST, *n* = 25; CDT, *n* = 25). Central donepezil infusion was discontinued at 120 days because of difficulties with repeatedly replenishing the microinfusion pump (Fig. 1b). The effect of central donepezil infusion on the growth of rats was evaluated by the body weight at 80 days post-MI. The rats were inspected daily, and gross postmortem examinations were conducted on the dead rats. The cause of death was classified as pump failure if edema, extreme weight loss accompanied by panting over the 24-h period prior to death, or pleural effusion was observed; otherwise, the cause of death was classified as sudden cardiac death.

### Statistical analysis

All statistical analyses were performed using Prism 7 (GraphPad, CA, USA). All values are expressed as mean ± standard error of the mean (SEM). For hemodynamic measurements, differences between the CST and CDT groups were tested using the unpaired Student’s *t*-test. Differences in HR and MBP before and during the treatment within each group were examined using a one-way analysis of variance (ANOVA) with repeated measures and post hoc Dunnett’s tests. For neurohumoral, capillary density, and biochemical data, the non-parametric Mann–Whitney *U*-test was used to compare the differences between the CST and CDT groups. Survival data are presented as Kaplan–Meier curves in the CST and CDT groups and the effect of treatment on 160-day survival was analyzed using a log-rank test. The differences were considered statistically significant when the *P*-value was < 0.05.

## Results

### Telemetric long-term hemodynamic measurements in conscious CHF rats

**Fig. 2:**
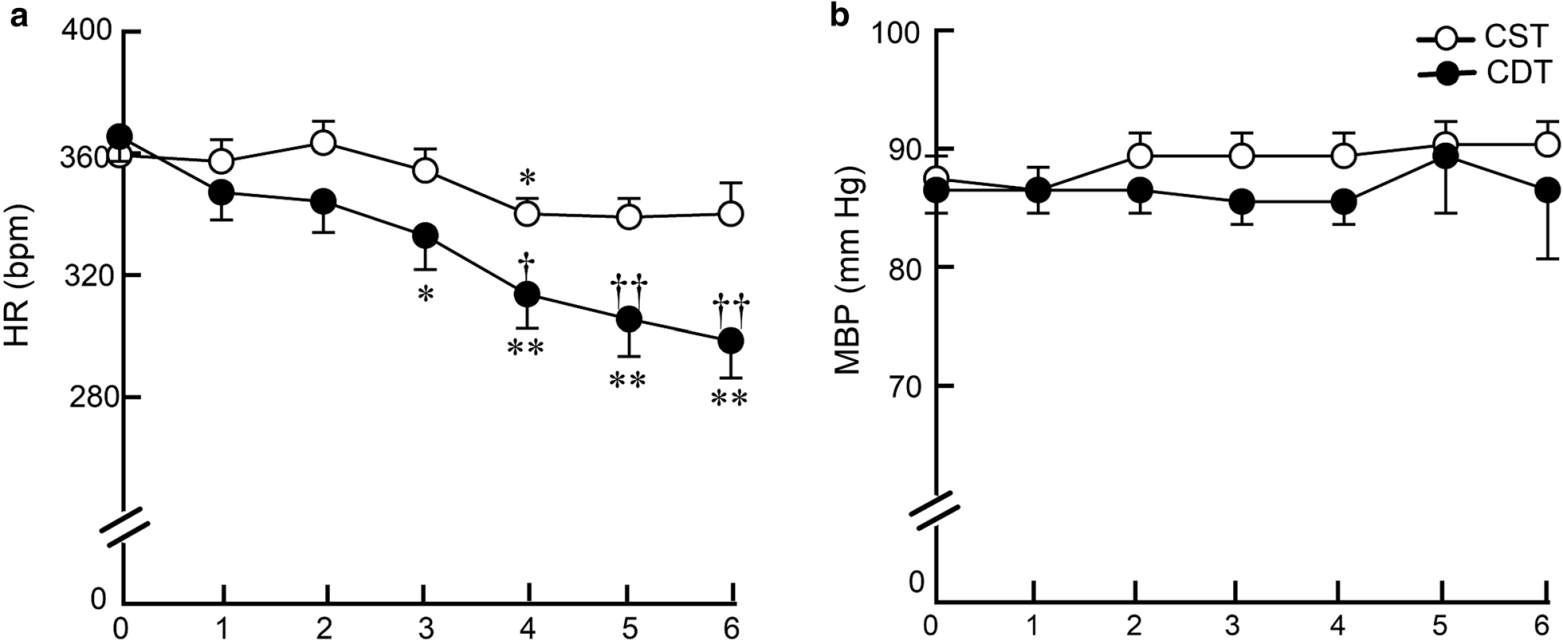
Effects of 6-week donepezil on telemetry hemodynamics. **a** Weekly averaged heart rate (HR), **b** weekly averaged mean blood pressure (MBP) in CST and CDT rats. Each point represents the average of 1-week continuous data from all animals in each group (CST, *n* = 13; CDT, *n* = 14). HR decreased significantly from the 3rd week of the treatment in the CDT group, whereas MBP did not change. Values are means ± SEM. ^†^*P* < 0.05, ^††^*P* < 0.01 in CDT vs. CST; *P < 0.05, **P < 0.01 vs. pretreatment values (week 0) of each group by post hoc Dunnett’s test

In the remodeling study, a telemetry device was used to accurately and continuously measure long-term hemodynamics in conscious CHF rats. The weekly average HR significantly and progressively decreased from the third week of treatment in the CDT group. The difference in HR between CDT and CST groups reached approximately 40 bpm during the 6th week of treatment (300 ± 12 vs. 341 ± 10 bpm, *P* < 0.01) (Fig. 2a). By contrast, there was no difference in the weekly average MBP between the CDT and CST groups (86 ± 6 vs. 90 ± 2 mm Hg, *P* = NS) (Fig. 2b).

**Fig. 3:**
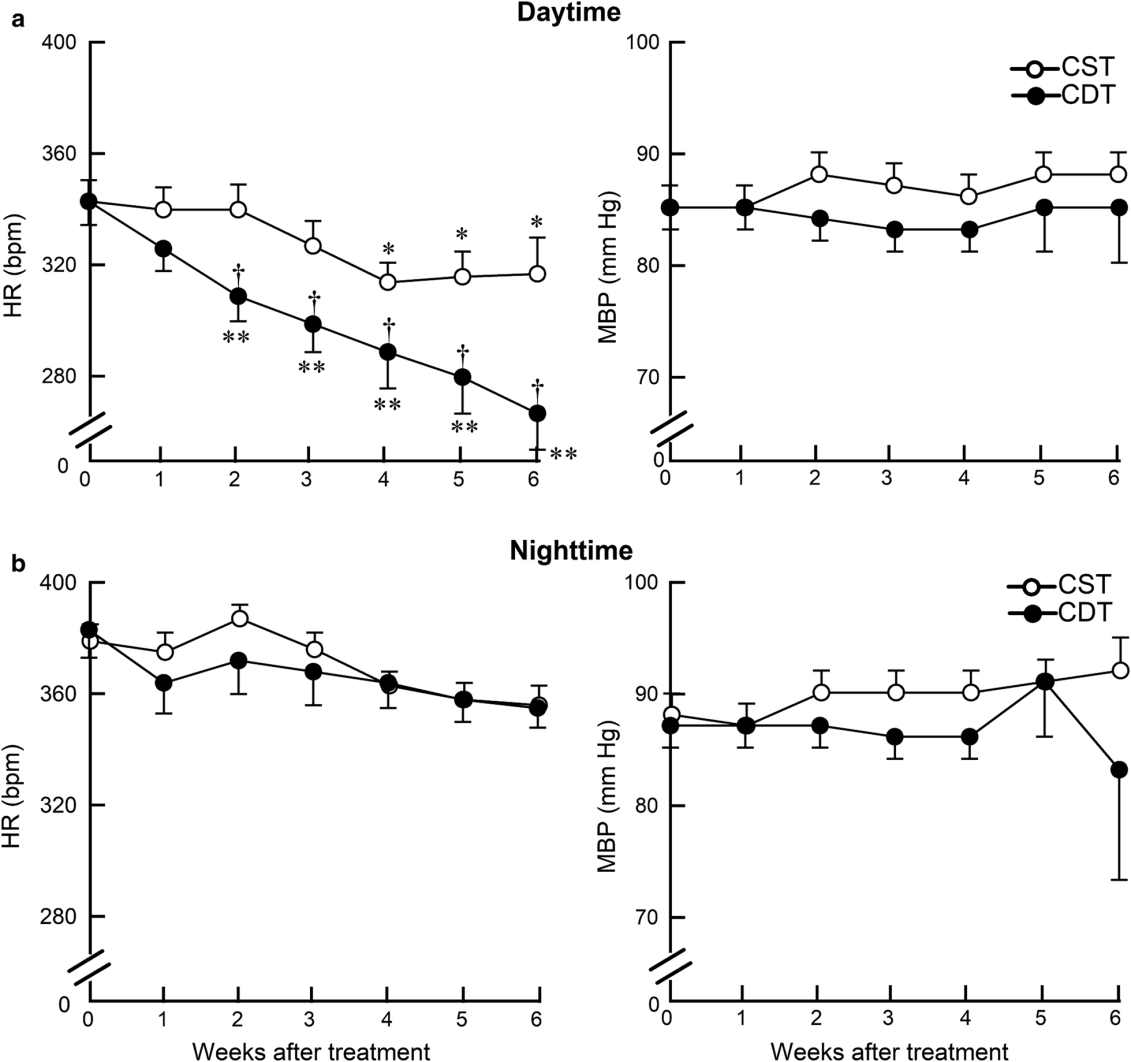
Effects of 6-week donepezil treatment on circadian telemetry hemodynamics. **a** Heart rate (HR) and mean blood pressure (MBP) during the daytime (at rest) in CST (*n* = 13) and CDT (*n* = 14) groups. **b** HR and MBP during the nighttime (active period) in CST (*n* = 13) and CDT (*n* = 14) groups. CDT group had a significant decrease in the daytime, but not in the nighttime HR compared to the CST group. Values are means ± SEM. ^†^*P* < 0.05, CDT vs. CST; *P < 0.05, **P < 0.01 vs. pretreatment values (week 0) of each group by post hoc Dunnett’s test

We further analyzed diurnal variation in HR. The difference in the daytime (6:00–18:00) HR between the CDT and CST groups reached approximately 50 bpm during the 6th week of treatment (267 ± 13 vs. 317 ± 13 bpm, *P* = 0.01) (Fig. 3a), but the nighttime (18:00–6:00) HR was not significantly different between the two groups (355 ± 7 vs. 356 ± 7 bpm, *P* = NS) (Fig. 3b). The difference between the daytime and the nighttime HR in the 4th week was 45 ± 2 bpm in the CST group; whereas, it was 75 ± 4 bpm in the CDT group (a 67% increase, *P* < 0.05). There was no significant difference of MBP between the CDT and CST groups (daytime, 85 ± 5 vs. 88 ± 2 mm Hg, *P* = NS, Fig. 3a; nighttime, 83 ± 10 vs. 92 ± 3 mm Hg, *P* = NS, Fig. 3b).

### Hemodynamic measurement under anesthesia, cardiac remodeling, and fibrosis

**Fig. 4:**
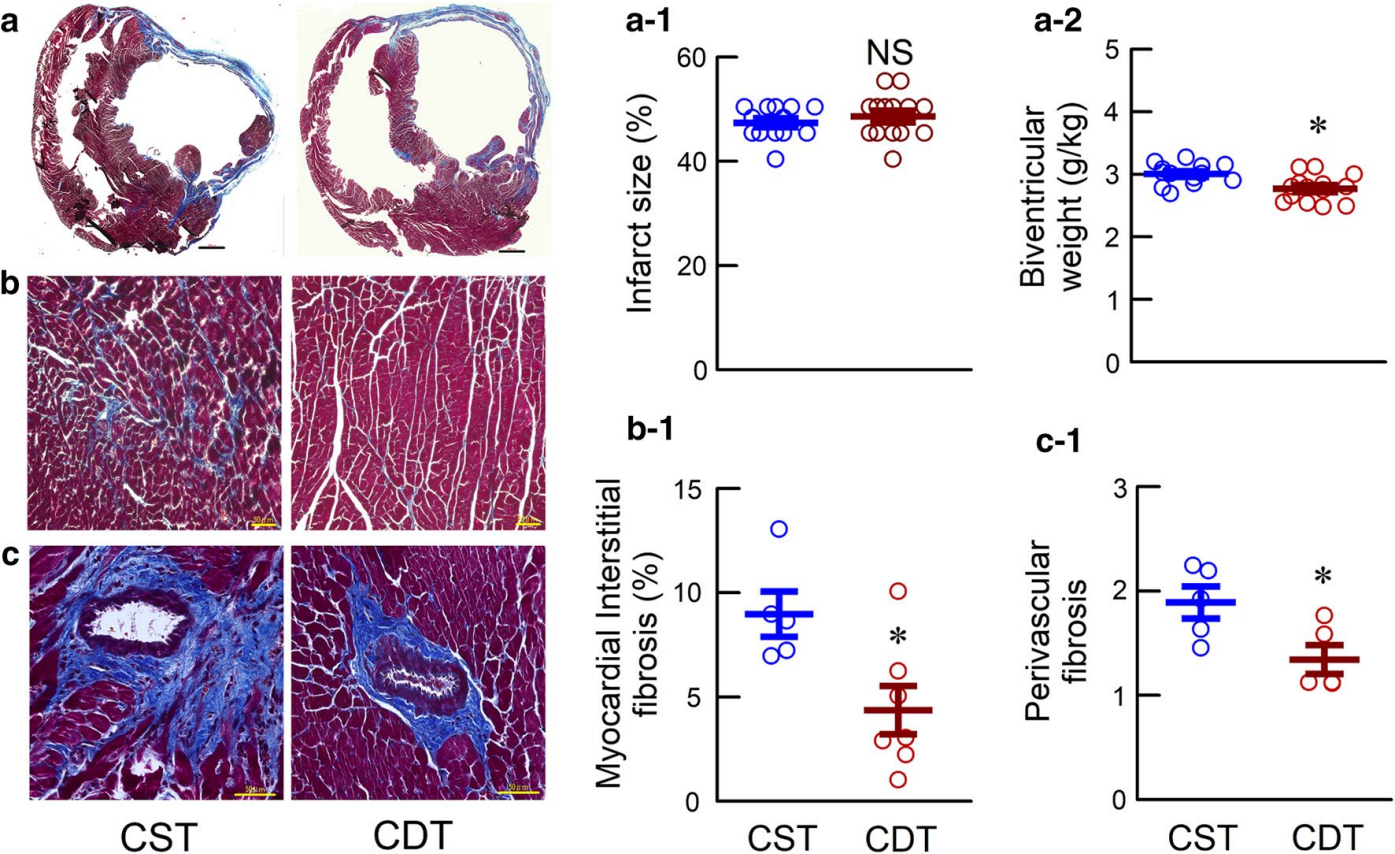
Masson’s trichrome staining of the ventricles in the chronic heart failure rats. **a** Representative biventricular sections of extensive myocardial infraction (MI) in the CST and CDT groups, scale bar: 300 µm. **a-1** Shown are infarct size (CST, *n* = 13; CDT, *n* = 14). **a-2** Biventricular weight normalized by body weight (CST, *n* = 13; CDT, *n* = 14). **b** Cardiac fibrosis, scale bar: 50 µm. **b-1** Myocardial interstitial fibrosis index in non-infarcted area (CST, *n* = 5, 20 fields; CDT, *n* = 7, 28 fields; each point is the mean value of 4 fields per animal). **c** Perivascular fibrosis, scale bar: 50 µm. **c-1** Perivascular fibrosis index in remote area (CST, *n* = 5, 14 fields; CDT, *n* = 5, 11 fields; each point is the mean value of 2–3 fields per animal). Values are means ± SEM. *NS* not significant, *P < 0.05 in CDT vs. CST by unpaired Student’s *t*-test

We initiated treatments 2 weeks after creating permanent MI. Specifically, MI was fixed, resulting in no significant difference in the MI size between the two groups (Fig. 4a-1, Table 1). In the remodeling study, hemodynamics under anesthesia, cardiac remodeling, and fibrosis in CHF rats after 8-week post-MI are shown in Fig. 4 and Table 1. Unlike in a conscious state, HR is not decreased in CDT than CST during anesthesia. CDT rats had significantly higher CI, LV + d*p*/d*t*_max_, LV − d*p*/d*t*_max_, lower LVEDP and RAP than CST rats. Prevention of cardiac dysfunction in CDT rats was accompanied by a significant prevention of cardiac hypertrophy as assessed by the normalized biventricular weight (Fig. 4a-2, Table 1) and by suppression of myocardial interstitial (4.51 ± 0.82 vs. 9.04 ± 0.92%, *P* < 0.05) (Fig. 4b-1), and perivascular fibrosis (1.37 ± 0.16 vs. 1.87 ± 0.17, *P* < 0.05) (Fig. 4c-1).

**Table 1:** Hemodynamics under anesthesia and plasma neurohumoral parameters in the remodeling study

	CST group (*n* = 13)	CDT group (*n* = 14)	*P* value
BW, g	428 ± 9	430 ± 7	NS
HW, g/kg	2.97 ± 0.05	2.77 ± 0.07	< 0.05
Infarct size, %	47 ± 1	48 ± 1	NS
MBP, mmHg	88 ± 2	83 ± 2	NS
HR, bpm	304 ± 8	309 ± 5	NS
CI, mL/min/kg	145 ± 11	181 ± 13	< 0.05
LV + d*p*/d*t*_max,_ mmHg/s	3116 ± 145	3699 ± 81	< 0.01
LV − d*p*/d*t*_max,_ mmHg/s	2682 ± 119	3108 ± 118	< 0.05
LVEDP, mmHg	28 ± 1	20 ± 2	< 0.01
RAP, mmHg	8 ± 1	5 ± 1	< 0.01
Plasma (pg/mL)			
Norepinephrine	1566 ± 217	741 ± 215	< 0.05
Epinephrine	2248 ± 445	893 ± 233	< 0.05
BNP	429 ± 27	359 ± 8	< 0.05
AVP	724 ± 49	490 ± 43	< 0.01
ANG II	138 ± 33	60 ± 10	< 0.05

Values are means ± SEM. *NS* not significant. Hemodynamic parameters assessed by unpaired Student’s *t*-test. Plasma neurohumoral parameters assessed using a non-parametric Mann–Whitney *U*-test

BW, body weight; HW, biventricular weight normalized by body weight; MBP, mean arterial pressure; HR, heart rate; CI, cardiac index (cardiac output/BW); LV + d*p*/d*t*_max_, maximum positive d*p*/d*t* of left ventricular pressure; LV − d*p*/d*t*_max_, maximum negative d*p*/d*t* of left ventricular pressure; LVEDP, left ventricular end-diastolic pressure; RAP, right atrial pressure. BNP, brain natriuretic peptide; AVP, arginine vasopressin; ANG II, angiotensin II

### Neurohumoral measurements

Table 1 shows the effects of central donepezil infusion on plasma neurohumoral factors in CHF rats after a 6-week treatment. Compared to CST rats, CDT rats had lower levels of plasma catecholamine, BNP, AVP, and ANG II.

### Immunohistochemical analysis

**Fig. 5:**
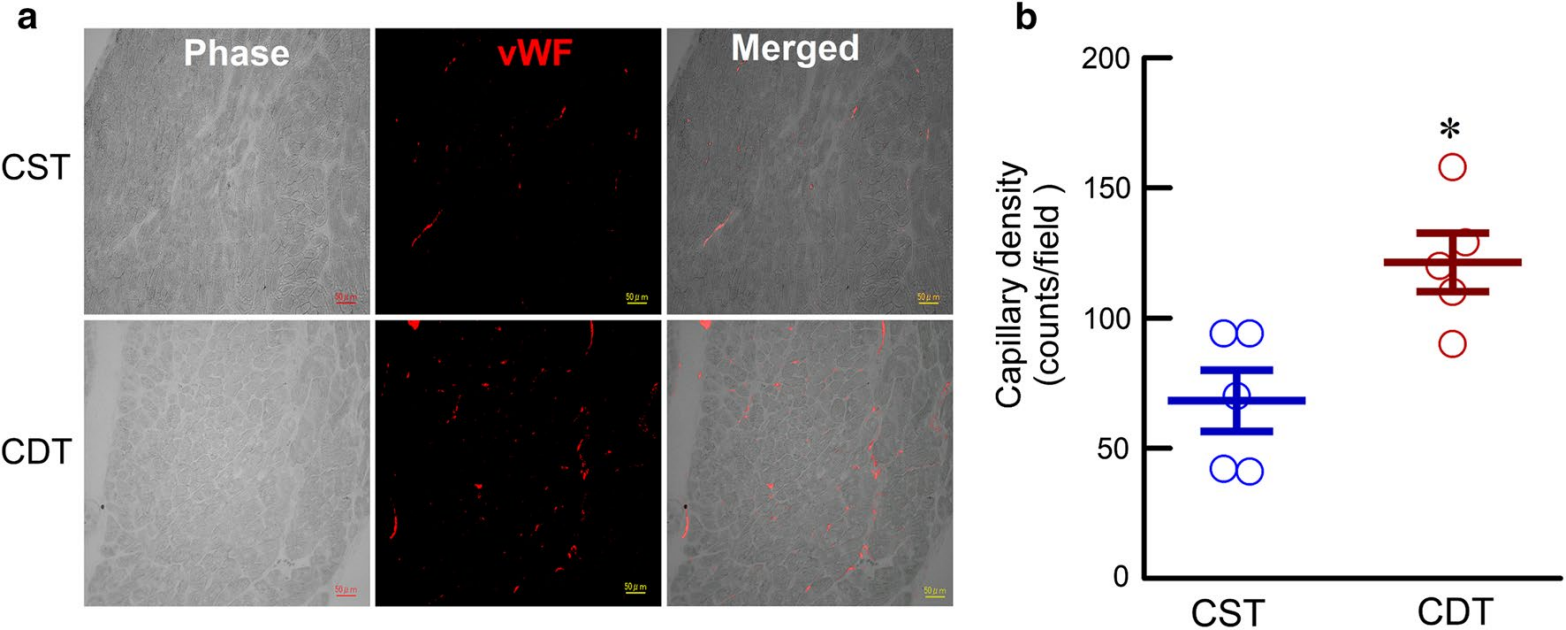
Immunohistochemical analysis in the peri-infarct areas of chronic heart failure rats. **a** Representative micrographs of von Willebrand factor (vWF, red) immunostaining. Scale bar: 50 µm. **b** Quantitative analysis of capillary density (CST, *n* = 5, 40 fields; CDT, *n* = 5, 40 fields, each point is the mean value of 8 fields per animal). Values are means ± SEM. *P < 0.05 in CDT vs. CST using a non-parametric Mann–Whitney *U*-test

Immunohistochemical study on the vWF revealed increased angiogenesis in the CDT group than in the CST group (Fig. 5a). The quantitative analysis demonstrated that capillary density was significantly higher in the CDT than in the CST group (121 ± 8 vs. 68 ± 11 cells/field, *P* < 0.05) (Fig. 5b).

### Survival study

**Fig. 6:**
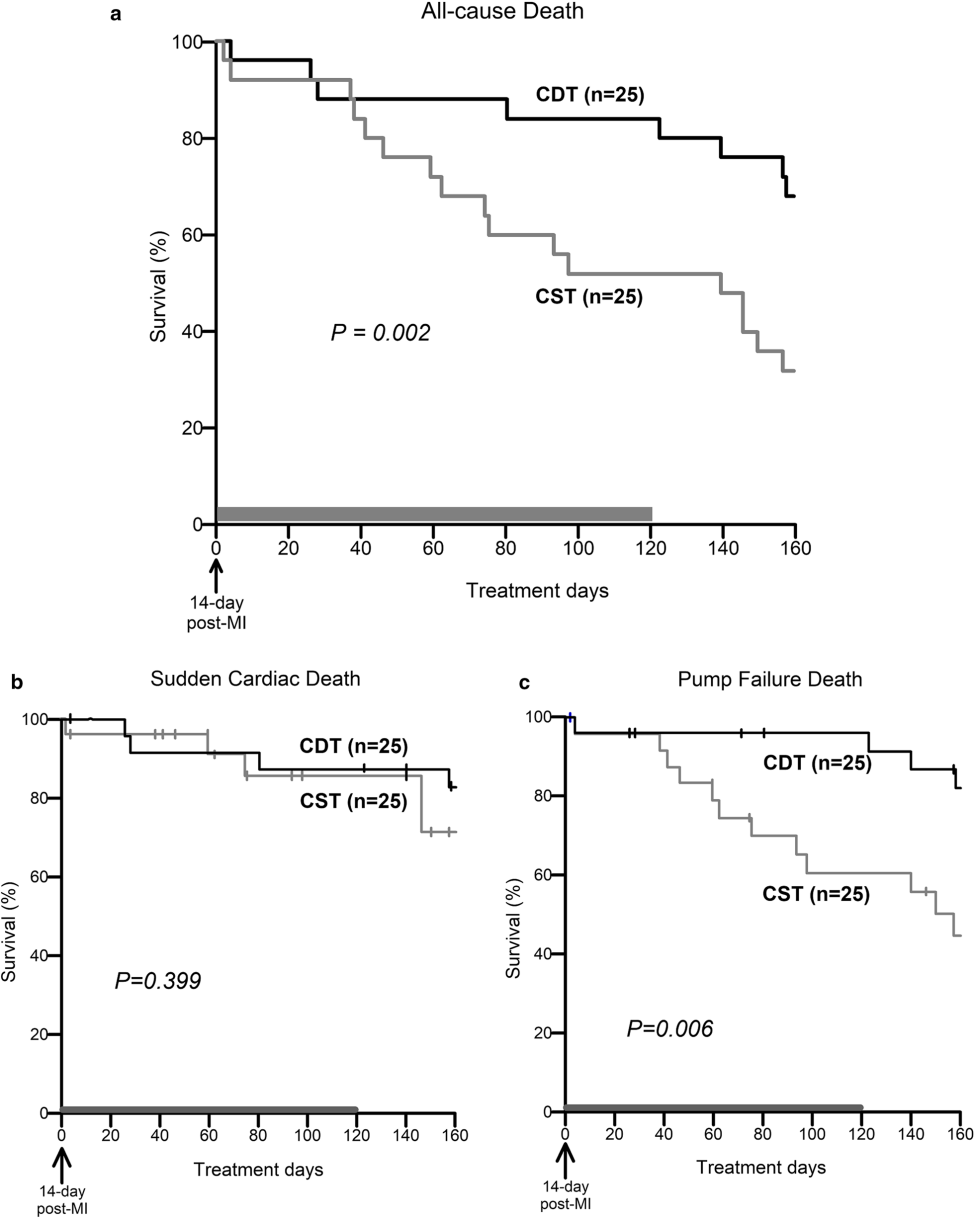
Kaplan–Meier survival curves of rats treated with central infusions of saline (CST, gray line, *n* = 25) and central infusions of donepezil for 120 days (CDT, black line, *n* = 25). Treatments were started 14 days post-myocardial infarction (MI). **a** Survival free from all-cause deaths in the CDT group significantly improved compared to the CST group (68% vs. 32%, *P* = 0.002). The median survival in the CST group was 99 days and was 181 days in the CDT group. **b** There were no differences in survival free from sudden cardiac deaths between the CDT and CST groups (80% vs. 70%, *P* = 0.399). **c** CDT markedly increased survival free from pump failure deaths (80% vs. 40%, *P* = 0.006). Vertical tick marks showed censored rats by pump failure death (in **b**) or sudden cardiac death (in **c**)

Fifty extensive MI-induced CHF rats were enrolled in the survival study. There was no difference in body weight between the surviving CST (*n* = 18) and CDT (*n* = 22) rats at 80 days post-MI (540 ± 10 vs. 534 ± 10 g, *P* = NS). The CDT markedly suppressed all-cause mortality. The 160-day survival rate was 68% in the CDT group and 32% in the CST group (Fig. 6a, *P* = 0.002). The median survival in the CST group was 99 days, compared to 181 days in the CDT group. Central donepezil infusion therapy achieved a 53% [(68–32)/68] reduction in the relative risk ratio of all-cause death. As shown in Fig. 6b, c, although there were no differences in sudden cardiac deaths between the CST and CDT groups (Fig. 6b, 80% vs. 70%, *P* = 0.399), the CDT markedly improved survival free from pump failure deaths (Fig. 6c, 80% vs. 40%, *P* = 0.006).

## Discussion

The major findings of the present study are beneficial effects of central donepezil microinfusion, which include (1) significantly reduced HR, especially resting HR; (2) suppressed catecholamine, BNP, AVP, and ANG II levels in plasma; (3) prevention of the progression of cardiac remodeling and dysfunction, and (4) improvement of the 160-day survival rate.

### Effects of central donepezil infusion therapy on HR reduction

Telemetric HR under non-stressful, conscious state revealed that the average HR reduction in the CDT group relative to the CST group was 40 bpm (Fig. 2a). The HR is decreased mainly during the daytime, i.e., at rest, and the difference reached approximately 50 bpm at the 6th week of treatment (Fig. 3a), but not during the nighttime, i.e., the active period (Fig. 3b). The difference between the daytime and the nighttime HR at the 4th week was 45 ± 2 bpm in the CST group; whereas, it reached 75 ± 4 bpm in the CDT group (a 67% increase, *P* < 0.05). The circadian variation of HR progressively increased in the CDT group (almost to a similar level with healthy animals) compared to the CST group. Both decreased sympathetic drive and increased parasympathetic efferent discharge appear to contribute to HR reduction, based on decreased plasma norepinephrine and epinephrine levels in the CDT group and increased high-frequency HR variability in the CHF rats when treated orally with donepezil [25]. Cardiac vagal efferent nerve activation reduces HR by direct excitation of muscarinic receptors on sinus node cells and by inhibiting norepinephrine release from sympathetic nerve endings [26]. Even though HR is a proxy marker for the treatment of CHF [27], resting HR is central to cardiac output, the clinical importance of higher resting HR in cardiovascular diseases as an independent risk factor of mortality and re-hospitalization conforms to our results [28, 29]. Therefore, the bradycardic effect seen in the CDT group may be an important factor in preventing cardiac dysfunction.

The bradycardic effect of donepezil may reproduce beneficial and important effects induced by β-blockers in the treatment of patients with CHF. However, because inhibition of β-receptors on cardiac myocytes would suppress myocardial contractility, β-blockers may not be suitable for patients with decompensation or pre-existing myocardial dysfunction. Maintenance of cardiac output in such patients depends partly on increased sympathetic drive [30]. Although possible vagus nerve activation may reduce ventricular contractility via suppression of a sympathetic effect, donepezil increased the CI, which suggests that the negative effect was limited.

Current treatments also focus on strategies that use selective HR-lowering agents, such as ivabradine, in patients with CHF [31–33]. A prolonged cardiac cycle is beneficial for enhancing and maintaining cardiac function by decreasing myocardial oxygen consumption, increasing coronary flow, and increasing ventricular filling volume [34]. Since ACh antagonizes the effects of β-adrenergic stimulation [35], donepezil may be an alternative to β-blockers for patients with severe CHF.

### Effects of central donepezil infusion therapy on cardiac remodeling and survival

In the CDT, cardiac remodeling and dysfunction were markedly prevented (Fig. 4a–c, Table 1) compared to the CST group. In the CDT rats, the plasma levels of catecholamine and AVP were significantly reduced. These results were consistent with our previous studies with orally administered donepezil [13, 14]. In addition, the CDT group showed significantly reduced plasma levels of ANG II and BNP compared to the CST group (Table 1), which may result from the decreased sympathetic outflow.

As per protocol, a primary endpoint of 160-day all-cause death was evaluated (i.e., at 174 days after MI, Fig. 6a). By 120 days of central donepezil infusion, the 160-day survival was markedly improved. We have gained a similar extent of improvement in survival as with oral donepezil [13, 14]. Further secondary analysis indicated that there was no significant difference in sudden cardiac deaths between CST and CDT groups (Fig. 6b), but in CDT pump failure deaths were markedly reduced (Fig. 6c). These results suggest that central donepezil infusion treatment beginning 2 weeks after MI mainly exerted its beneficial effect by preventing the progression of cardiac remodeling and dysfunction.

Collectively, central donepezil infusion appears to reproduce most but not all of the cardioprotective effects elicited by orally administered donepezil. Because the concentration of donepezil in brain tissue was not measured in the present study or our previous oral administration study [13], the efficacy of donepezil could be different between the two studies. Further, there was an additional burden of central infusion instrumentation in the present study, which might have influenced the severity of CHF. With these discrepancies kept in mind, numerical comparisons are as follows: the average HR reduction relative to the untreated group (40 bpm in the CDT group vs. 30 bpm in the oral administration group), the improvement of 140-day survival rate (27 vs. 25%), the suppression of cardiac hypertrophy (7 vs. 11%) and plasma catecholamine (NE, 741 vs. 497 pg/ml; Epi, 893 vs. 495 pg/ml) [13]. Although the targeted region is unknown, orally administered donepezil reaches the central nervous system [21–23], and likely increase cardiac vagal efferent discharge to exert cardioprotective effects. According to previous reports, a peripherally acting acetylcholinesterase inhibitor, pyridostigmine, also exerts beneficial effects in CHF [11, 17, 36, 37]. This implies that oral donepezil may not only act centrally, but may also potentially act through peripheral pathways.

### Probable mechanisms involved in donepezil treatment

To the best of our knowledge, this study is the first to confirm the central mechanisms contributing to the beneficial effects of donepezil in CHF. In the present study, rats of the CDT group exhibited reduced HR compared to that of the CST group. This indicated that continuous central donepezil microinfusion suppressed central acetylcholinesterase activity and increased central ACh levels [21–23], which in turn exerted bradycardic effects by decreasing sympathetic outflow, increasing parasympathetic tone, or both. Meanwhile, central donepezil infusion significantly promoted angiogenesis (Fig. 5). The α7-nicotinic ACh receptor (α7-nAChR) distributes widely in central and peripheral neuronal or non-neuronal tissue and is involved in the cholinergic anti-inflammatory reflex [38]. Local or systemic inflammation information is conveyed to the central nervous system via vagal afferent fibers and then increases vagal efferent output to modulate an inflammatory reaction and angiogenesis via peripheral α7-nAChR [39–41].

### Possible clinical implications

This study indicates that drug delivery to the central nervous system would be a method to modulate (peripheral) autonomic nervous balance [42, 43] and that correcting autonomic imbalance may result in cardioprotective effects [12, 44]. The direct intracerebroventricular infusion is by no means necessary. Rather, orally administered donepezil likely exerts its beneficial effects by reaching the central nervous system through the blood–brain barrier [21–23]. Similar drugs that reach the central nervous system to modulate autonomic nervous activities may be promising measures for the treatment of CHF.

## Limitations

In this study, an extensive MI-induced CHF rat model was used in consideration of the precarious clinical condition of patients with severe CHF with MI and requiring hospitalization. However, the experimental animals were young and may have had a reserve of autonomic function that could be adjusted by various therapeutic interventions. In contrast, patients with severe or end-stage CHF may have limited responsive capacity. In addition, because clinical trials are usually conducted in patients of CHF with various pharmacological treatment backgrounds, it would be difficult to determine the performance of single-drug treatment. It may be an important factor in translating this basic study outcome to the clinical studies. Since we did not assess brain tissue to specify the central action site of donepezil, an exact mechanism for the cardioprotective effect of centrally administered donepezil remains unclear. Direct evidence for the increased vagal efferent activity during central donepezil microinfusion awaits further studies.

## Conclusions

Central microinfusion of donepezil seemingly reproduced the cardioprotective benefits exerted by oral administration that were previously observed in CHF rats. Although the findings of the current study leave far more uncertainty regarding the mechanisms of donepezil’s benefits, it is probable to conclude that pharmacologically increased central ACh levels may restore the balance of autonomic function, which involves the regulation of inflammation. Therefore, we propose that the central modulation of autonomic function by donepezil is a potential novel therapeutic strategy for patients with CHF.

## Data Availability

The datasets used and/or analyzed during the current study are available from the corresponding author on reasonable request.

## Abbreviations

CHF: Chronic heart failure
MI: Myocardial infarction
ACh: Acetylcholine
MBP: Mean blood pressure
HR: Heart rate
CST: Central saline treatment
CDT: Central donepezil treatment
LVEDP: Left ventricular end-diastolic pressure
LV + d*p*/d*t*_max_: Maximum positive d*p*/d*t* of left ventricular pressure
LV − d*p*/d*t*_max_: Maximum negative d*p*/d*t* of left ventricular pressure
RAP: Right atrial pressure
CI: Cardiac index
BNP: Brain natriuretic peptide
AVP: Arginine vasopressin
ANG II: Angiotensin II
vWF: von Willebrand factor
